# Neurobiology Informed, Mobile Technology Augmented Interventions for Late-Life Depression and Suicidality

**DOI:** 10.1093/geroni/igaa057.2117

**Published:** 2020-12-16

**Authors:** George Alexopoulos

**Affiliations:** Weill Medical College of Cornell University, White Plains, New York, United States

## Abstract

Depression is a major risk factor of suicide in late life. Evidence based psychotherapies for late-life depression are underutilized, mainly because of their complexity. In response, we created “Engage”, an innovative streamlined psychotherapy that relies on neurobiology findings to identify core behavioral pathology of late-life depression and targets it with simple cognitive-behavioral strategies of known efficacy, co-designed with community therapists so that its interventions can be mastered my community based clinicians. We demonstrated that “Engage” is non-inferior to the evidence based Problem Solving Therapy and documented that behavioral activation precedes improvement of depression. We have also shown that activities with important others have the strongest behavioral activation value. “Engage” served as a model for three novel, mobile technology augmented psychotherapies that address the clinical and psychosocial context of distinct populations of depressed older adults. Our interventions and mobile apps provide interventions for emotion regulation as a way of reducing suicidality.

